# Supplementary material for: Guinea grass (Megathyrsus maximus) agronomic performances in mixed cultivation using irrigation condition, North-Western Ethiopia
Source: PLoS One. 2025 Feb 6;20(2):e0316565. doi: 10.1371/journal.pone.0316565 (PMC11801539; doi:10.1371/journal.pone.0316565)
Supplement: S1 File — (PDF) [file pone.0316565.s001.pdf]

S1 file. Raw data collected from plant morphology and dry matter yield per ha (DOCX)

Block	SP	HA	PH	TNPP	NLPP	LLPP	NRPP	RLPP	LSR
B1	SP20	HA60	51	35.35	249.25	18.41	76.19	5.90	1.48
B1	SP20	HA90	64	41.30	352.00	20.30	94.45	6.33	1.39
B1	SP20	HA120	79	55.20	389.00	26.30	101.67	6.66	1.23
B1	SP30	HA60	59	36.90	257.00	19.30	77.30	5.72	1.67
B1	SP30	HA90	67	42.50	361.00	21.30	96.40	6.20	1.45
B1	SP30	HA120	91	50.32	387.00	25.35	102.23	6.70	1.20
B1	SP40	HA60	57	34.70	269.00	19.15	78.30	6.12	1.72
B1	SP40	HA90	75	45.30	364.00	22.43	95.40	6.43	1.39
B1	SP40	HA120	81	51.70	402.00	27.25	107.60	6.90	1.32
B2	SP20	HA60	49	36.20	243.60	17.60	74.60	5.30	1.46
B2	SP20	HA90	71	41.60	359.60	22.10	94.90	6.04	1.36
B2	SP20	HA120	80	50.30	401.60	25.90	103.20	6.90	1.24
B2	SP30	HA60	61	37.80	249.30	18.90	75.20	5.60	1.64
B2	SP30	HA90	71	43.80	372.50	23.40	98.10	6.40	1.39
B2	SP30	HA120	87	51.90	405.70	27.60	106.30	7.03	1.29
B2	SP40	HA60	67	38.40	257.40	19.30	78.90	5.80	1.71
B2	SP40	HA90	74	44.60	367.20	23.80	97.30	6.50	1.39
B2	SP40	HA120	93	53.60	411.30	29.30	109.80	7.30	1.32
B3	SP20	HA60	48	35.90	246.20	17.81	73.90	4.90	1.42
B3	SP20	HA90	72	40.80	362.80	22.80	93.90	6.30	1.41
B3	SP20	HA120	83	50.60	398.70	25.30	102.40	7.20	1.30
B3	SP30	HA60	63	34.10	251.20	18.40	74.90	5.70	1.69
B3	SP30	HA90	76	44.10	371.90	24.10	97.60	6.70	1.43
B3	SP30	HA120	89	51.90	406.46	26.90	107.20	7.63	1.31
B3	SP40	HA60	66	39.20	258.70	19.80	77.10	5.90	1.80
B3	SP40	HA90	76	45.20	369.30	24.60	98.10	6.40	1.60
B3	SP40	HA120	94	53.20	415.30	30.20	112.10	8.30	1.20
