# Supplementary material for: Guinea grass (Megathyrsus maximus) agronomic performances in mixed cultivation using irrigation condition, North-Western Ethiopia
Source: PLoS One. 2025 Feb 6;20(2):e0316565. doi: 10.1371/journal.pone.0316565 (PMC11801539; doi:10.1371/journal.pone.0316565)
Supplement: S2 File — (PDF) [file pone.0316565.s002.pdf]

S2 file. Raw data on chemical composition analysis of Guinea grass

Block	SP	HA	DM%	OM%	CP%	NDF%	ADF%	ADL%	Ash%
B1	SP20	HA60	89.34	86.3	17.19	71.87	42.33	10.68	13.7
B1	SP20	HA90	90.67	85.65	15.93	79.84	46.51	11.05	14.35
B1	SP20	HA120	91.58	84.88	11.47	88.14	49.62	12.38	15.12
B1	SP30	HA60	93.48	85.64	19.14	68.64	40.82	10.2	14.36
B1	SP30	HA90	94.17	84.86	16.82	78.16	43.4	10.31	15.14
B1	SP30	HA120	94.8	83.69	12.16	81.59	47.83	11.79	16.31
B1	SP40	HA60	95.28	83.39	20.97	65.64	32.6	9.61	16.61
B1	SP40	HA90	95.66	83.72	18.52	76.41	39.6	10.97	16.28
B1	SP40	HA120	95.76	83.12	13.81	77.66	44	11.4	16.88
B2	SP20	HA60	90.01	87.11	17.63	71.22	43.2	10.24	12.89
B2	SP20	HA90	92.11	86.39	15.24	78.15	47.15	11.07	13.61
B2	SP20	HA120	92.044	85.1	11.54	80.07	52.69	12.78	14.9
B2	SP30	HA60	93.9	85.67	19.85	70.31	40.3	9.96	14.33
B2	SP30	HA90	94.52	85.09	16.53	76.28	41	10.18	14.91
B2	SP30	HA120	94.7	84.99	12.14	79.2	44.76	11.21	15.01
B2	SP40	HA60	96.82	84.45	20.34	58.1	33.9	8.77	15.55
B2	SP40	HA90	96.03	84.15	18.63	69.61	39.7	9.43	15.85
B2	SP40	HA120	96.71	83.78	13.23	77.3	41.35	10.91	16.27
B3	SP20	HA60	90.68	87.04	17.1	67.25	42.27	10.41	12.96
B3	SP20	HA90	91.33	85.92	15.84	74.74	47.21	11.08	14.08
B3	SP20	HA120	92.5	85.45	11.1	85.8	50.99	12.39	14.55
B3	SP30	HA60	93.61	84.27	19.94	65.19	39.48	9.8	15.73
B3	SP30	HA90	94.04	84.1	16.64	73.17	41.74	10.42	15.9
B3	SP30	HA120	94.19	83.78	12.9	81.1	44.66	11.69	16.22
B3	SP40	HA60	95.058	83.19	20.79	61.27	33.57	8.61	16.81
B3	SP40	HA90	96.32	82.64	18.45	68.12	36.55	9.57	17.36
B3	SP40	HA120	97.59	81.55	13.97	77.03	39.47	10.25	18.45
