# Supplementary material for: Guinea grass (Megathyrsus maximus) agronomic performances in mixed cultivation using irrigation condition, North-Western Ethiopia
Source: PLoS One. 2025 Feb 6;20(2):e0316565. doi: 10.1371/journal.pone.0316565 (PMC11801539; doi:10.1371/journal.pone.0316565)
Supplement: Attachment — Submitted filename: Response to Reviewers(2024).docx [file pone.0316565.s003.docx]

**Dear Editor,**

I would like to thank you for giving us feedback to improve our MS through your committed work and the anonymous reviewers’ efforts.

We have gone through your comments and revised accordingly and prepared an author’s response below. We also well come further comments to qualify the manuscript.

| comments , suggestion and recommendations from PLOS ONE | Authors response |
| --- | --- |
| **Academic Editor** |  |
| 1. When submitting your revision, we need you to address these additional requirements.    Please ensure that your manuscript meets PLOS ONE's style requirements, including those for file naming | Thank you for your information. It accepted and included to revised manuscript. |
| 2. In your Methods section, please provide additional information regarding the permits you obtained for the work. Please ensure you have included the full name of the authority that approved the field site access and, if no permits were required, a brief statement explaining why. | Thank you for your comment; we have included our statement regarding the field site access in the MM section. |
| 3. In the online submission form, you indicated that [Datasets are available at the hand of corresponding author].  All PLOS journals now require all data underlying the findings described in their manuscript to be freely available to other researchers, either 1. In a public repository, 2. Within the manuscript itself, or 3. Uploaded as supplementary information. | Thank you for your requesting raw data, any stage we can do it, when the manuscript accepted for publication, Within the manuscript itself. |
| **Reviewer's Responses to Questions No.1 to5** | Thank you for your response per questions, I understand status of the manuscript, it is updated to meet the points. |
| **5. Review Comments to the Author** |  |
| **Reviewer #1:** |  |
| **General comments**  1. In the abstract there must be research objectives, research methods, results and discussion, and the originality of the research. | Thank you for your comments, it accepted and incorporated |
| 2. The introduction should be concise and clear. Other than that, in quoting, you must use a new language and not copy and paste. | Thank you for your comments, it is revised |
| 3. The research method must be clear about the research location, research methods that are in accordance with the research theory. | Thank you for your comments, it is updated |
| 4. The results and discussion must be detailed, clear, concise, concise, and easy to understand. | Thank you for your concerns, it is checked and revised in the manuscript |
| 5. Conclusions must be short and clear, provide research suggestions for further researchers | Thank you for your suggestion , it accepted and revised |
| 6. Must follow the format that is in accordance with the submitted journal. | Thank you for your suggestion, it is updated per format PLOS ONE |
| Specific comments |  |
| 1. Change the botanical name of guinea grass from Panicum maximum to Megathyrsus maximus. | Thank you for you corrections, it corrected |
| 2. Use a similar format for keywords.  3. Confirm the statistical design—whether it is CRBD (Completely Randomized Block Design) or RCBD (Randomized Complete Block Design). | Thank you for you corrections, it corrected |
| 4. Table 4: CP values are on the slightly higher side. Please check. | thank you for your concerned, it checked the raw data and traced back the data from the technical |
| 5. Include the soil fertility status of the experimental field. | Thank you for your recommendation, at the time of the experiment, it was not sampled and analyzed due to budget constrain. |
| **Reviewer #2:** |  |
| Thank you for giving me the opportunity to review your manuscript entitled "Guinea grass (Panicum Maximum) Agronomic Performance for Mixed Cultivation using Irrigation conditions, Northern Western Ethiopia". I very much enjoyed reading your manuscript. The author’s purpose that at 40 cm plant spacing with 90 day's harvest age is better for nutritional value of Guinea grass however maximum other parameters are given better result with 120 day's harvest age.  The manuscript is well written. | Thank you for your comments |
| It's may be typing mistake in 43 & 281 which is Sliver Leaf Desmodium actually it Silver Leaf Desmodium.  Discussions are logical with tables and figures. | Thank you for you corrections, it corrected |
| References are suitable for this manuscript, I think if 8, 10, 13,16,17,19,& 23 references are same style as like 2 it will be better. | Thank you for your suggestion, it is updated per format PLOS ONE, some of them are follow as citation recommendations form journal and reports format where it is accessed. |
