# Supplementary material for: Guinea grass (Megathyrsus maximus) agronomic performances in mixed cultivation using irrigation condition, North-Western Ethiopia
Source: PLoS One. 2025 Feb 6;20(2):e0316565. doi: 10.1371/journal.pone.0316565 (PMC11801539; doi:10.1371/journal.pone.0316565)
Supplement: Attachment — Submitted filename: Response to Reviewers(2025).docx [file pone.0316565.s005.docx]

**Dear Editor,**

I would like to thank the academic editor of PLOS ONE and the anonymous reviewers for providing us with suggestions that assisted us in improving the content of our paper. We have incorporated your comments, amended them, and prepared the response shown in the table below. We are happy to receive further comments, thank you in advance.

| comments , suggestion and recommendations from PLOS ONE | | Authors response |
| --- | --- | --- |
| **Academic Editor,** PLOS ONE | |  |
| line17 to 19, page 1 | | Thank you for your respected notes, it is removed and corrected |
| line 37 and 39, page2 | | Thank you for your corrections, the space is corrected |
| line57 to 61, page 3 | | Thank you for feedback, it is rewritten |
| line 66,page 3 | | Thank you for your corrections, the space is corrected |
| line 71 to72, page 4 | | Thank you for your correction, it is done |
| line 79 and 121 page 5 to6 | | Thank for your valuable editing and corrections, it is done |
| line 136,page7 | | Thank you for your insightful comments. As stated in the methodology section, the legume is utilized as an axillary service, and we simply offer the grass's outcomes to achieve the goals. |
| line237, page 13 | | Thank you for correction, it is done |
| **Journal Requirements: reference list**  **page 17 to 21** | list of authors name in each reference list used in the manuscript | It conforms to the PLOS ONE guideline, which states that references with more than six authors should list the names of the first six authors first, then "et al." |
|  | Reference list of 1,4, 11,15,16,17,19,20,23,27 | The format has been updated, and the used prefix http:// has been deleted from doi. |
|  | Reference number 7,8, | It is corrected, and its web availability is given according to the format. |
|  | Reference number 9 | The citation format is available, but the PDF document has been retracted and reason is not available on the web repository.; <http://ir.bdu.edu.et/handle> |
|  | Reference number 14 | It has been updated to reflect the format of the online-accessible |
|  | Reference 12 | page number is included as of format |
| **Reviewer's Responses to Questions** | | Thank you for your response to the queries; I attempt to improve based on your valuable feedback; it has been modified to address the points. |
